# Supplementary material for: Ca2+-Triggered (de)ubiquitination Events in Synapses
Source: Mol Cell Proteomics. 2025 Mar 13;24(4):100946. doi: 10.1016/j.mcpro.2025.100946 (PMC12008530; doi:10.1016/j.mcpro.2025.100946)
Supplement: Supplementary material [file mmc7.pdf]

Supplementary material

Supplementary figures:

Figure S1: Glutamate release assay and proteome changes in depolarized synaptosomes under different stimuli. 3

Figure S2: K-ε-GG peptide enrichment specificity. 4

Figure S3: Ubiquitination sites mapped at selected proteins. 5

Figure S4: Comparison of log_2 fold changes (log2FC) between the TMT and PRM quantitative MS methods. 5

Figure S5: Post-translational modifications mapped at the regulatory domain of CaMKIIα and their abundance changes in depolarized synaptosomes under different stimuli. 7

Figure S6: CaMKII structures in the closed/autoinhibited and open states. 8

Supplementary Tables:

Table S1: Enriched GO biological function terms of all identified ubiquitinated proteins according to SynGO database: 9

Table S2: Enriched GO biological function terms of regulated ubiquitinated proteins according to SynGO database: 9

Table S3: Enriched GO biological function terms of all identified ubiquitinated proteins according to ShinyGO database 9

Table S4: Selected K-ε-GG peptides for targeted PRM analysis in depolarized synaptosomes: 10

Table S5: Selected peptides for the absolute quantification analysis of CaMKIIα in synaptosomes: 11

Table S6: Selected peptides for the absolute quantification analysis of CaMKIIα in stimulated HeLa (Kyoto) cells: 11

Table S7: Ubiquitinated E3 ligases in depolarized synaptosomes 12

Table S8: Ubiquitinated DUBs in depolarized synaptosomes. 12

Supplementary data:

Suppl. Data 1: TMT reporter ion intensities used for the assessment of regulated ubiquitination site in EGTA-vs-Ca2+ treated synaptosomes........ 13

Suppl. Data 2: All modifications, precursors masses and m/z ratios observed for each ubiquitinated peptide........ 13

Suppl. Data 3: Method parameters of PRM analysis 13

Suppl. Data 4: Light to heavy ratios of targeted peptides used for the assessment of regulated ubiquitination sites in EGTA -vs- Ca2+ treated synaptosomes by PRM. 13

Suppl. Data 5: Light to heavy ratios of targeted CaMKIIa peptides used for the assessment of changes in K291 ubiquitination and T286 phosphorylation of CaMKIIα in EGTA -vs- Ca2+ treated synaptosomes by PRM. 13

Suppl. Data 6: Light to heavy ratios of targeted peptides used for the assessment of changes in K291 ubiquitination and T286 phosphorylation of CaMKIIα in DMSO-vs-ionomycin treated HeLa (Kyoto) cells. 13

1. Supplementary figures

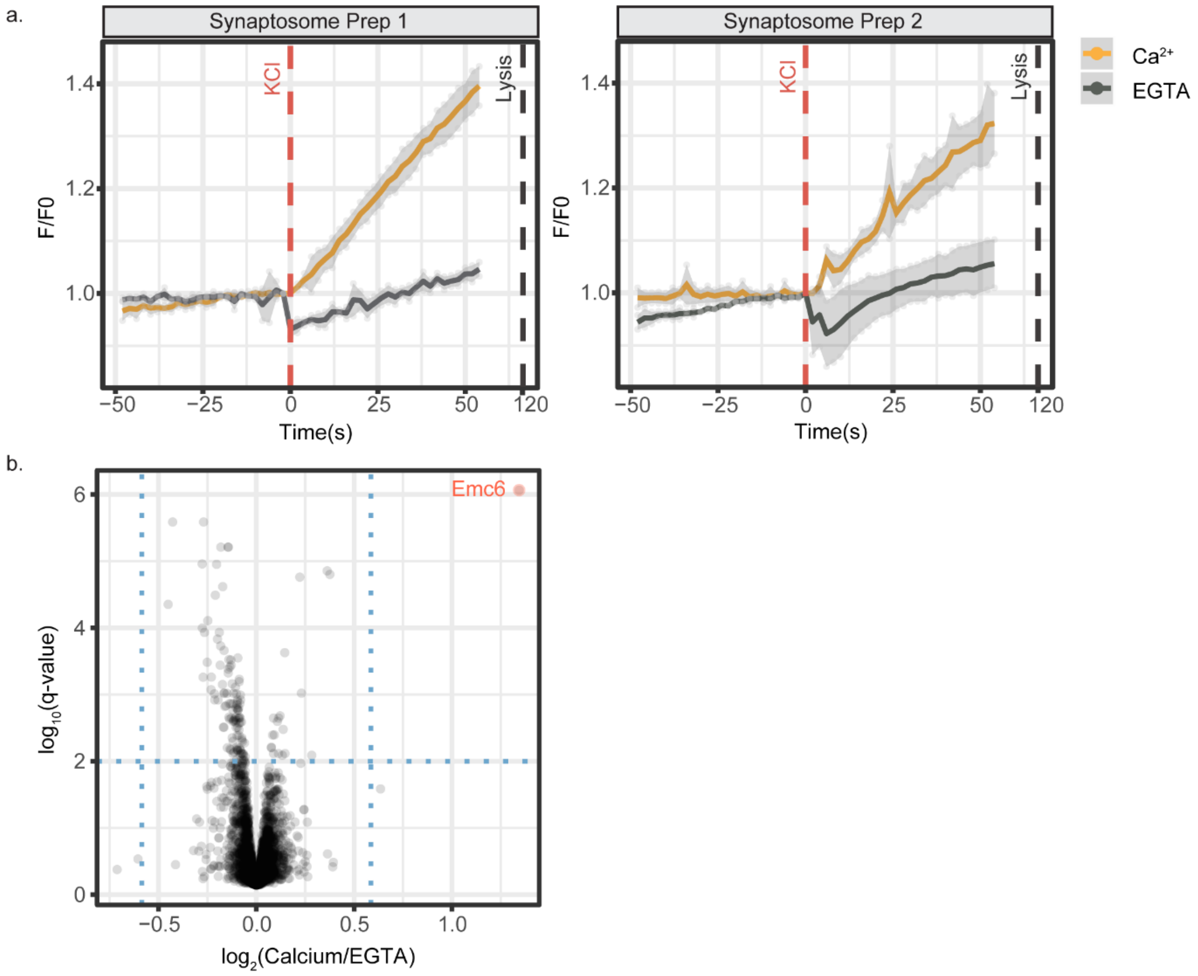

Figure S1: Glutamate release assay and proteome changes in depolarized synaptosomes under different stimuli. *a) KCl depolarization of synaptosomes in the presence of* Ca^{2+} *or EGTA as a control to account for* Ca^{2+}*-independent exocytosis. Glutamate release was monitored in response to chemical depolarization in real time (x-axis) and correlated with normalized fluorescence intensity (F/F0) (y-axis). Yellow line corresponds to the average response of synaptosomes depolarized in the presence of* Ca^{2+} *from three independent stimulation experiments. Grey line corresponds to the average response of synaptosomes depolarized in the presence of EGTA from three independent stimulation experiments. Shaded grey areas represent the standard deviation at each time point .b) Volcano plot showing* log_2*(intensity fold change) of proteins quantified under* Ca^{2+} *vs. EGTA conditions against* $-log_{10}$*(q-value). Proteins that show at least an abundance change of 1.5-fold at 1% FDR are coloured in red.*

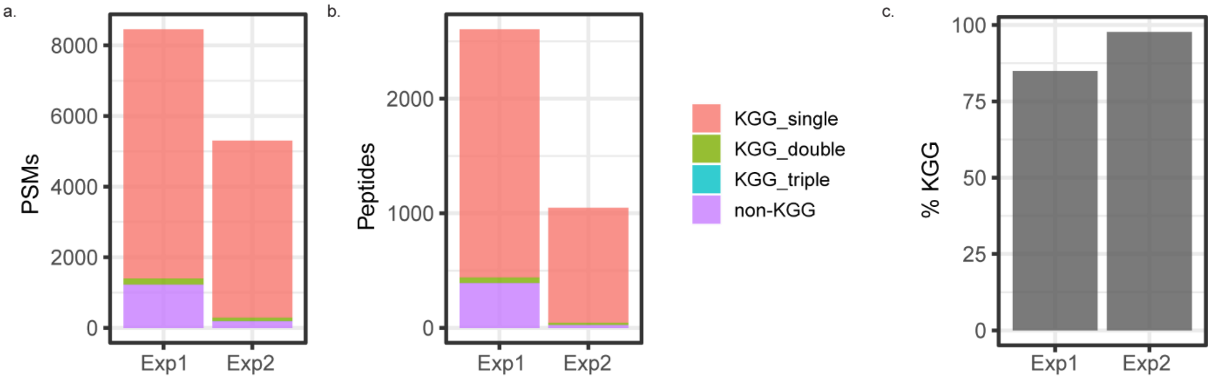

Figure S2: K-ε-GG peptide enrichment specificity. *a) the number of peptide-spectrum matches (PSMS) per experiment. b) The number of peptides identified per experiment. The different colours correspond to the number of ubiquitination remnants (K-ε-GG) mapped at individual peptides. c) The percentage of K-ε-GG peptides that was identified per experiment. Exp1: Experiment 1, Exp2: Experiment 2*

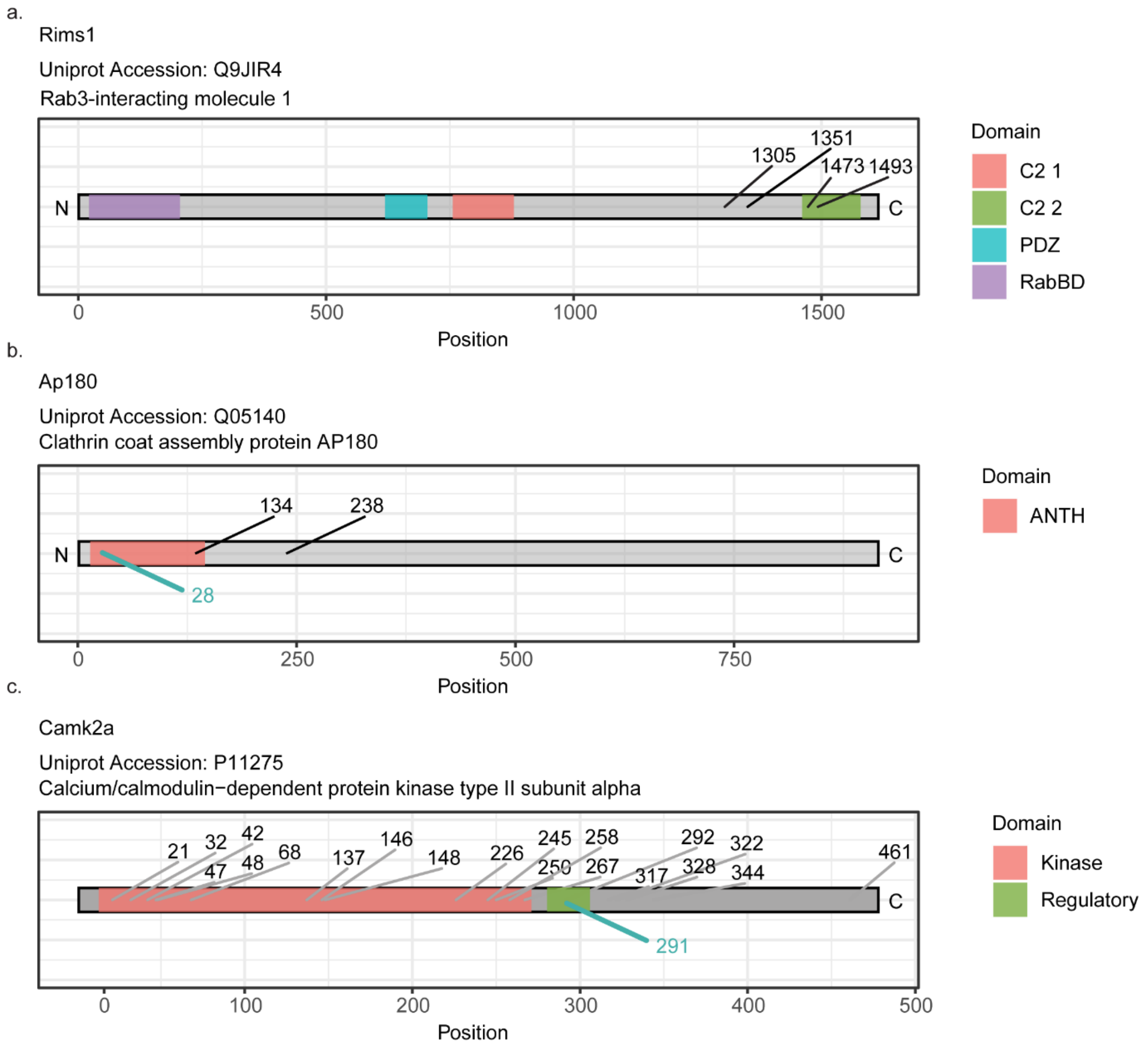

Figure S3: Ubiquitination sites mapped at selected proteins. *The protein sequence is shown as a grey bar and the corresponding domains are color-coded. The exact position of the ubiquitination sites with PEP <0.01 as determined by MaxQuant**(1, 2)** are shown. The ubiquitination sites not affected by Ca^{2+} influx are shown in black, whereas those sites that show abundance changes in response to Ca^{2+} influx are highlighted in cyan blue. a, b) The domains of Rims1 and Ap180 are presented as annotated in UniProt**(3)**, whereas c) the domains of CaMKIIα are manually annotated according to Chao et al.**(4)**.*

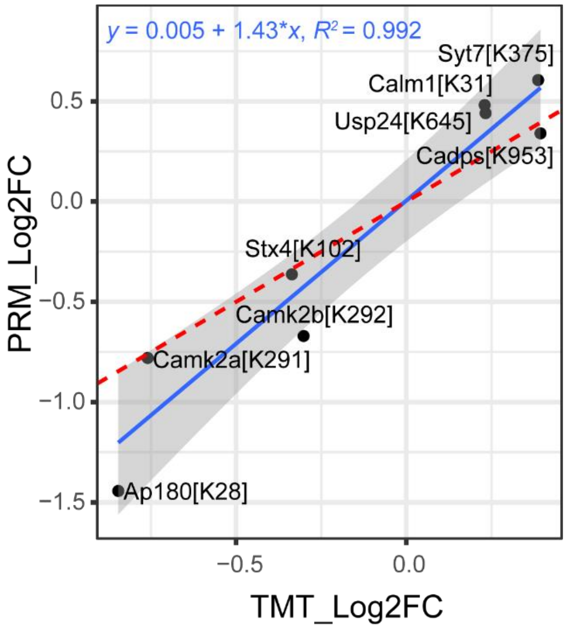

Figure S4: Comparison of log_2 fold changes (log2FC) between the TMT and PRM quantitative MS methods. *The regression line, its equation and the R2 value are highlighted in blue colour. The red dotted line represents the theoretical y=x relationship, indicating identical log_2FC values between the two methods. Each data point corresponds to a ubiquitinated peptide quantified by both methods.*

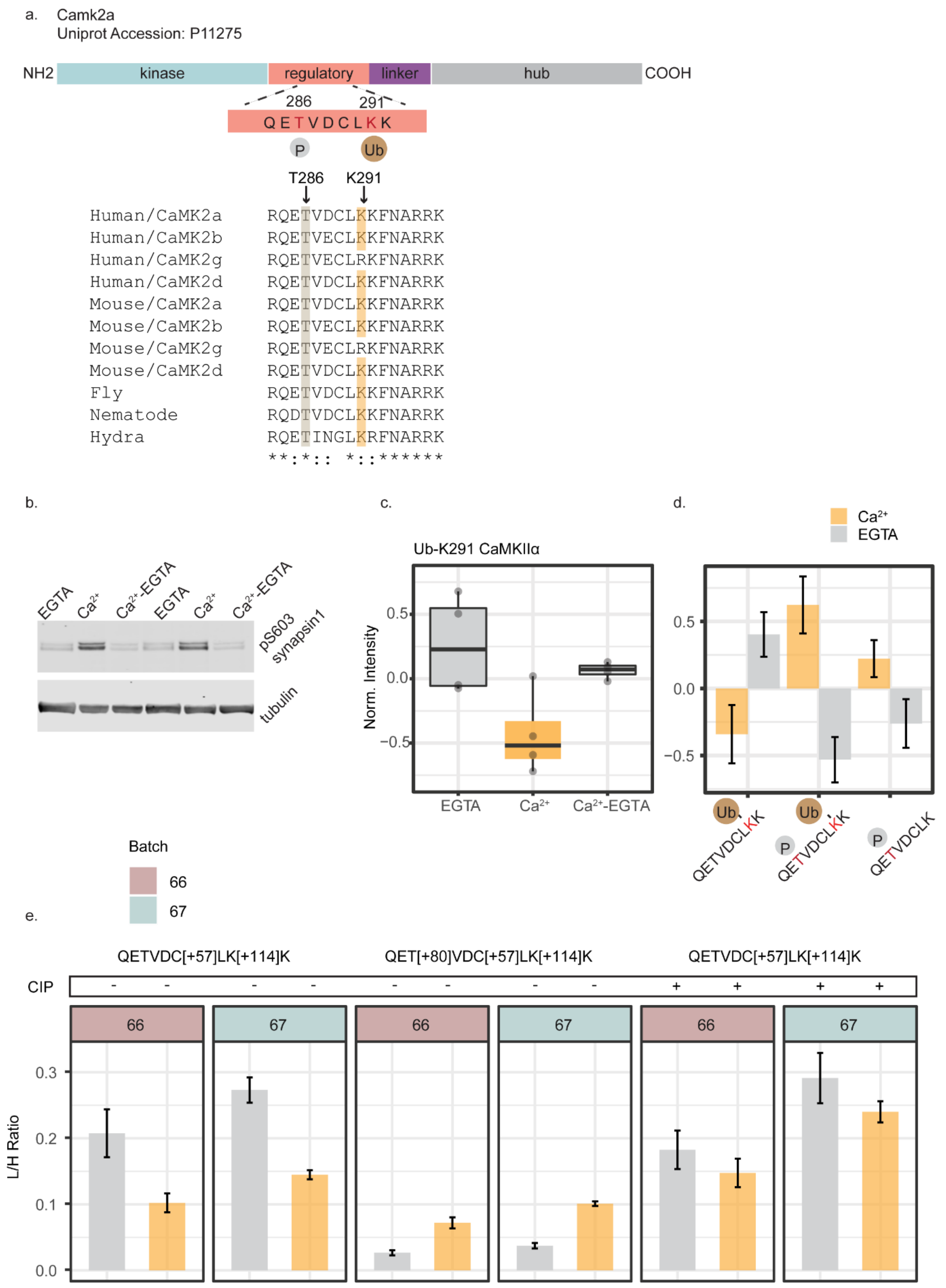
a.
Camk2a
Uniprot Accession: P11275
NH2
kinase
regulatory
linker
hub
COOH
286
291
QETVDCLKK
P
Ub
T286
K291
Human/CaMK2a RQETVDCLKKFNARRK
Human/CaMK2b RQETVECLKKFNARRK
Human/CaMK2g RQETVECLRKFNARRK
Human/CaMK2d RQETVDCLKKFNARRK
Mouse/CaMK2a RQETVDCLKKFNARRK
Mouse/CaMK2b RQETVECLKKFNARRK
Mouse/CaMK2g RQETVECLRKFNARRK
Mouse/CaMK2d RQETVDCLKKFNARRK
Fly RQETVDCLKKFNARRK
Nematode RQDTVDCLKKFNARRK
Hydra RQETINGLKRFNARRK
:*:: *::****
b.
EGTA
Ca2+
Ca2+-EGTA
EGTA
Ca2+
Ca2+-EGTA
pS603 synapsin1
tubulin
c.
Ub-K291 CaMKIIα
Norm. Intensity
0.5
0.0
−0.5
EGTA
Ca2+
Ca2+-EGTA
d.
Ca2+
EGTA
0.5
0.0
−0.5
Ub
QETVDCLKK
Ub
P
QETVDCLKK
P
QETVDCLK
e.
Batch
66
67
QETVDC[+57]LK[+114]K
QET[+80]VDC[+57]LK[+114]K
QETVDC[+57]LK[+114]K
CIP
- - - - - - - - + + + +
66
67
66
67
66
67
L/H Ratio
0.3
0.2
0.1
0.0

Figure S5: Post-translational modifications mapped at the regulatory domain of CaMKIIα and their abundance changes in depolarized synaptosomes under different stimuli. *Protein sequence alignment of the regulatory domain of CaMKII homologues was performed using the multiple sequence alignment tool T-Coffee*(5)*. The regulatory T286 and K291 (numbering based on human CaMKIIα with UniprotID Q9UQM7) are highlighted with grey and yellow colours, respectively. b) SDS/PAGE and immunoblotting against phosphosynapsin and tubulin (as a control for gel loading) were performed. An increase in the phosphorylation state of synapsin was observed in the presence of* Ca^{2+}*, indicative of functional synaptosomes. Upon* Ca^{2+} *chelation the phosphorylation level of synapsin decreased and returned to basal levels. (c) A bottom-up proteomic workflow was used for the TMT-based quantification of formerly ubiquitinated (K-ε-GG) peptides. Boxplot shows K-ε-GG modified peptide corresponding to ubiquitinated CaMKIIα at K291. The x-axis corresponds to the different treatments of the synaptosomes and the y-axis corresponds to the normalized TMT reporter ion intensities. d) Peptide species corresponding to part of the regulatory region of CaMKIIα were detected either ubiquitinated on K291 or doubly modified by ubiquitination and phosphorylation under our experimental conditions. The figure shows log2-normalised reporter-ion intensities of differently modified peptide species derived from two independent TMT6 experiments. The phosphorylated peptide species corresponding to autophosphorylated CaMKIIα at T286 was included in the plot based on our previous quantification of the phosphorylation sites in resting and excited synaptosomes*(6)*. e) Barplot showing the mean light-to-heavy peak area ratios of modified CaMKIIα peptides before and after QuickCIP treatment from two different batches of synaptosomal preparation. Error bars correspond to the standard estimation estimated by three independent stimulation replicates.*

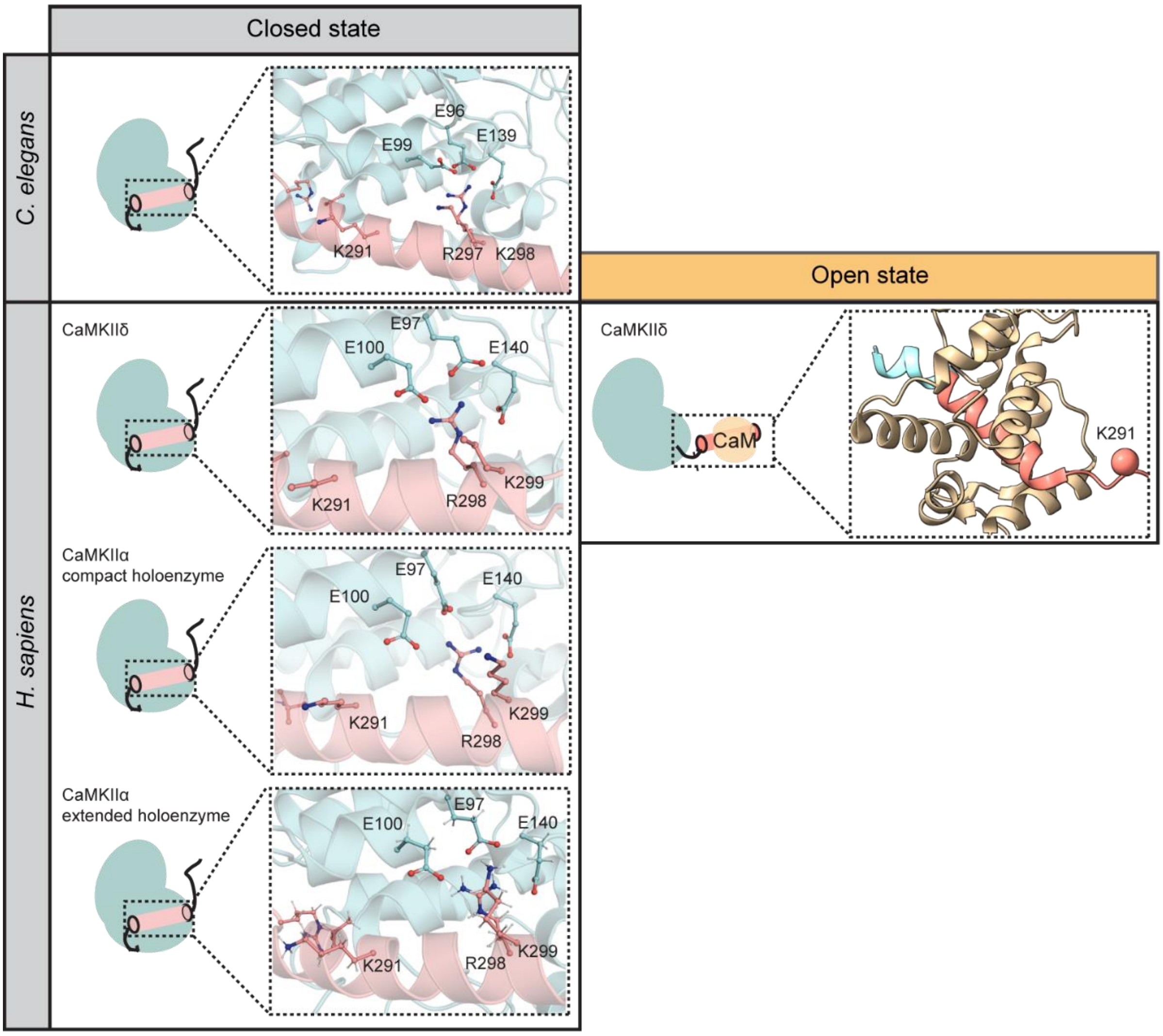

Figure S6: CaMKII structures in the closed/autoinhibited and open states. *X-ray structure of C. elegans CaMKII (UNC-43) in the autoinhibited state (PDB code 2bdw (7)). X-ray structure of human CaMKIIδ in the autoinhibited state (PDB code 2vn9 (8)). X-ray structure of one subunit of human CaMKIIa holoenzyme in the compacted autoinhibited state (PBD code 3soa (4)). Electron microscopy structure of one subunit of human CaMKIIa holoenzyme in the extended autoinhibited state (PBD code 5u6y (9)). X-ray structure of human CaMKIIδ in the open/active state (PDB code 2wel (8)). The kinase and regulatory/autoinhibitory domains of CaMKII are highlighted with cyan and salmon colours, respectively. The interactions between basic residues of the autoinhibitory domain with acidic residues of the catalytic site of the kinase domain are shown in the right panels. K291 of CaMKIIα or K292 of CaMKIIδ that is targeted for ubiquitination is also shown in all structures.*

2. . Supplementary tables

Table S1: Enriched GO biological function terms of all identified ubiquitinated proteins according to SynGO database: *A list of proteins with well-localized ubiquitination sites identified in Ca-vs-EGTA experiments was used as a foreground in pathway enrichment analysis using the SynGO database. GO biological functions terms that were significantly enriched at an enrichment FDR <0.01 are shown.*

GO term ID	GO term name - hierarchical structure	FDR
SYNGO:synprocess	process in the synapse	1.54E-14
SYNGO:presynprocess	├─ process in the presynapse	3.78E-10
GO:0099504	| ├─ synaptic vesicle cycle	4.97E-08
GO:0016079	| | ├─ synaptic vesicle exocytosis	0.0047
GO:0048488	| | └─ synaptic vesicle endocytosis	0.002181
SYNGO:postsynprocess	├─ process in the postsynapse	5.02E-05
GO:0099072	| ├─ regulation of postsynaptic membrane neurotransmitter receptor levels	0.001806
GO:0099536	├─ synaptic signaling	0.0047
GO:0099537	| ├─ trans-synaptic signaling	0.0047
GO:0050808	├─ synapse organization	0.002993

Table S2: Enriched GO biological function terms of regulated ubiquitinated proteins according to SynGO database: *A list of proteins with regulated ubiquitination sites as determined in Ca-vs-EGTA experiments was used as a foreground in pathway enrichment analysis using the SynGO database. GO biological functions terms that were significantly enriched at an enrichment FDR <0.01 are shown.*

GO term ID	GO term name - hierarchical structure	FDR
SYNGO:synprocess	process in the synapse	8.9972E-06
SYNGO:presynprocess	├─ process in the presynapse	1.45029E-07
GO:0099504	| ├─ synaptic vesicle cycle	3.50589E-07
GO:0016079	| | ├─ synaptic vesicle exocytosis	8.9972E-06
GO:0048488	| | └─ synaptic vesicle endocytosis	0.000366676
GO:0099525	| ├─ presynaptic dense core vesicle exocytosis	1.00844E-05
SYNGO:postsynprocess	├─ process in the postsynapse	0.005995807

Table S3: Enriched GO biological function terms of all identified ubiquitinated proteins according to ShinyGO database *A list of proteins with well-localized ubiquitination sites identified in Ca-vs-EGTA experiments was used as a foreground in pathway enrichment analysis using the ShinyGO database. GO biological functions terms that were significantly enriched at an enrichment FDR <0.005 are shown.*

Pathway	Enrichment FDR	Fold Enrichment
Synaptic vesicle cycle	5.19E-10	1.958419
Vesicle-mediated transport in synapse	1.06E-09	1.888838

Regulation of neurotransmitter levels	3.71E-08	1.854714
Neurotransmitter transport	1.88E-07	1.828719
Regulated exocytosis	2.59E-07	1.807157
Neurotransmitter secretion	4.97E-07	1.907698
Signal release from synapse	4.97E-07	1.907698
Potassium ion transport	1.43E-06	1.8335
Regulation of exocytosis	4.07E-06	1.79824
Synaptic vesicle exocytosis	3.72E-05	1.932437
Potassium ion transmembrane transport	9.29E-05	1.785838
Regulation of neurotransmitter transport	0.000118115	1.933845
Postsynapse organization	0.000140545	1.703963
Regulation of neuronal synaptic plasticity	0.000293933	2.2478
Regulation of synaptic plasticity	0.000342866	1.6405
Negative regulation of transmembrane transport	0.001226744	1.918123
Regulation of neurotransmitter secretion	0.001226744	1.883248
Regulation of proteasomal protein catabolic process	0.001475548	1.686625
Cytokinesis	0.001733313	1.79824
Cell-cell junction assembly	0.001733313	1.961716
Adenylate cyclase-modulating G protein-coupled receptor signaling pathway	0.001733313	1.835445
Negative regulation of ion transmembrane transport	0.001733313	1.986627
Negative regulation of ion transport	0.001733313	1.849618
Regulation of proteasomal ubiquitin-dependent protein catabolic process	0.002787001	1.804336
Glycolytic process	0.002791192	1.991897
Negative regulation of cation transmembrane transport	0.002791192	1.991897
ATP generation from ADP	0.002791192	1.991897
Synaptic vesicle recycling	0.002791192	1.964906
Negative regulation of neuron death	0.002839177	1.663914
Regulation of regulated secretory pathway	0.002839177	1.72631
Import across plasma membrane	0.003132779	1.743748
Negative regulation of neuron apoptotic process	0.004875467	1.717722
Cytoskeleton-dependent cytokinesis	0.004875467	1.918123
Regulation of proteolysis involved in cellular protein catabolic process	0.004937502	1.585387
Sodium ion transmembrane transport	0.004937502	1.72631

Table S4: Selected K-ε-GG peptides for targeted PRM analysis in depolarized synaptosomes: *26 K-ε-GG peptides representing 26 ubiquitination sites were selected for PRM analysis. The exact modified peptide sequence of the targets is presented; [+57] and [+114] correspond to the nominal masses of carbamidomethylation and K-ε-GG ubiquitin remnant, respectively.*

Protein	Position	Uniprot ID	Peptide sequence
CaMK2a	K291	P11275	QETVDC[+57]LK[+114]K
AP180	K28	Q05140	AVC[+57]K[+114]ATTHEVMGPK
Unc13c	K992	Q62770	ILAGDSSSVDEK[+114]AR
Caps1	K953	Q62717	TDYNLC[+57]NGK[+114]FHK
Caps1	K1249	Q62717	LQGVLDSTLNSK[+114]TYETIR

Syt7	K375	Q62747	IYLSWK[+114]SGPGEVK
Stx1b	K93	P61265	LK[+114]AIEQSIEQEEGLNR
Stx1a	K94	P32851	LK[+114]SIEQSIEQEEGLNR
Stx4	K102	Q08850	AQLK[+114]AIEPQK
Snap25	K76	P60881	EAEK[+114]NLTDLGK
Snap25	K103	P60881	K[+114]AWGNNQDGVVASQPAR
Vamp2	K59	P63045	DQK[+114]LSELDDR
Vamp7	K172	Q9JHW5	TENLVDSSVTFK[+114]TTSR
Ppap2b	K15	P97544	AIVPESK[+114]NGGSPALNNNPR
Pclo	K2766	D3Z9C7	LHSYVK[+114]AEEDPMEDPYELK
Calm2	K31	P0DP31	DGDGTITTK[+114]ELGTVMR
Usp24	K645	F1LSM0	SFIK[+114]QTYQK
Camk2b	K292	F1LNI8	QETVEC[+57]LK[+114]K
Usp5	K423	D3ZVQ0	ALIGK[+114]GHPEFSTNR
Ubc	K6	P62982	MQIFVK[+114]TLTGK
Ubc	K11	P62982	TLTGK[+114]TITLEVEPSDTIENVK
Ubc	K27	P62982	TITLEVEPSDTIENVK[+114]AK
Ubc	K29	P62982	AK[+114]IQDKEGIPPDQQR
Ubc	K33	P62982	IQDK[+114]EGIPPDQQR
Ubc	K48	P62982	LIFAGK[+114]QLEDGR
Ubc	K63	P62982	TLSDYNIQK[+114]ESTLHLVLR

Table S5: Selected peptides for the absolute quantification analysis of CaMKIIα in synaptosomes: *Eight peptides derived from CaMKIIα were monitored in depolarized synaptosomes. The exact modified peptide sequence of the targets is presented; [+57], [+80], and [+114] correspond to the nominal masses of carbamidomethylation, phopshorylation and K-ε-GG ubiquitin remnant, respectively.*

**Protein**	**Peptide sequence**
CaMKIIα	ITAAEALK
	ITQYLDAGGIPR
	QET[+80]VDC[+57]LK
	QET[+80]VDC[+57]LK[+114]K
	QETVDC[+57]LK
	QETVDC[+57]LK[+114]K
	RITAAEALK
	VLAGQEYAAK

Table S6: Selected peptides for the absolute quantification analysis of CaMKIIα in stimulated HeLa (Kyoto) cells: *Ten peptides derived from CaMKIIα WT and CaMKIIα K291R mutant variant were monitored in stimulated HeLa (Kyoto) cells. The exact modified peptide sequence of the targets is presented; [+57], [+80], and [+114] correspond to the nominal masses of carbamidomethylation, phopshorylation and K-ε-GG ubiquitin remnant, respectively.*

Protein	Peptide sequence
CaMKIIα	ITAAEALK
	ITQYLDAGGIPR
	QET[+80]VDC[+57]LK
	QET[+80]VDC[+57]LK[+114]K
	QETVDC[+57]LK
	QETVDC[+57]LK[+114]K
	RITAAEALK
	VLAGQEYAAK
CaMKIIα Mut	QET[+80]VDC[+57]LR
	QETVDC[+57]LR

Table S7: Ubiquitinated E3 ligases in depolarized synaptosomes

Uniprot ID	Gene name	Protein description	Sites
Q5PQN2	Bfar	Bifunctional apoptosis regulator	387
Q8CIN9	Rffl	E3 ubiquitin-protein ligase rififylin	258
Q66H79	Trim32	Tripartite motif protein 32	508;407;473;361
D3ZL82	Trim37	Tripartite motif protein 37 (Predicted)	411
D3ZJB8	Arih2	RBR-type E3 ubiquitin transferase	376
D3ZXL1	Arih1	RBR-type E3 ubiquitin transferase	142;437
Q5XIQ4	Mgrn1	E3 ubiquitin-protein ligase MGRN1	165;102
F1M8V2	Ube4b	Ubiquitination factor E4B	665
A0A0G2JVW5	Huwe1	HECT-type E3 ubiquitin transferase	2698;324
Q62940	Nedd4	E3 ubiquitin-protein ligase NEDD4	556;450;483
F1LRN8	Nedd4l	HECT-type E3 ubiquitin transferase	456;624;506;524;447

Table S8: Ubiquitinated DUBs in depolarized synaptosomes.

Uniprot ID	Gene name	Protein description	Sites
B2GUZ1	Usp4	Ubiquitin carboxyl-terminal hydrolase 4	835
D3ZPG5	Usp30	Ubiquitin carboxyl-terminal hydrolase 30	338;310
Q4VSI4	Usp7	Ubiquitin carboxyl-terminal hydrolase 7	870;421
Q6J1Y9	Usp19	Ubiquitin carboxyl-terminal hydrolase 19	836;843
Q5D006	Usp11	Ubiquitin carboxyl-terminal hydrolase 11	819;794
D3ZC84	Usp9x	Ubiquitinyl hydrolase 1	1627;1632;1811;112;1798;315;1435
F1LPJ7	Usp33	Ubiquitin carboxyl-terminal hydrolase	226
D3ZLQ8	Usp20	Ubiquitin carboxyl-terminal hydrolase	325;907
D3ZVQ0	Usp5	Ubiquitin carboxyl-terminal hydrolase	423;476;743;508;574;184;64;318;357;360;391;406;178;793;558;20
D4ACD3	Usp25	Ubiquitin specific protease 25 (Predicted)	251;631
A0A0G2JTF5	Usp34	Ubiquitinyl hydrolase 1	1317
A0A0G2JZU8	Usp9y	Ubiquitin-specific peptidase 9, Y-linked	1809
D3ZBB7	Usp32	Ubiquitinyl hydrolase 1	810
F1LSM0	Usp24	Ubiquitin-specific peptidase 24	418;1761;650;645

Q5U2N2	Usp14	Ubiquitin carboxyl-terminal hydrolase	448;342;238;300;214
Q00981	Uchl1	Ubiquitin carboxyl-terminal hydrolase isozyme L1	71;115;123;4
O35815	Atxn3	Ataxin-3	200
Q8CF97	Vcpip1	Deubiquitinating protein VCPIP1	181;385;763;710;690;697;1012;375;361;878;198;407;330;457;140
O88767	Park7	Parkinson disease protein 7 homolog	182;93;130;41
Q499N6	Ubxn1	UBX domain-containing protein 1	178;83;105
B2RYG6	Otub1	Ubiquitin thioesterase OTUB1	188;71;73
Q32Q05	Yod1	Ubiquitin thioesterase OTU1	252
D3ZH40	Otud7b	Ubiquitinyl hydrolase 1	263;93
Q66HP6	Spata2	Spermatogenesis-associated protein 2	27
Q8R424	Stambp	STAM-binding protein	107
Q5BJY4	Josd1	Josephin-1	180
D4ABZ4	Otud7a	Ubiquitinyl hydrolase 1	89
P46462	Vcp	Transitional endoplasmic reticulum ATPase	696;236;8;18;486;60;251;231;668;651;217;502;658
B2RYM5	Brcc3	Lys-63-specific deubiquitinase BRCC36	180

3. Supplementary data

Suppl. Data 1: *TMT reporter ion intensities used for the assessment of regulated ubiquitination site in EGTA-vs-Ca2+ treated synaptosomes*

Suppl. Data 2: *All modifications, precursors masses and m/z ratios observed for each ubiquitinated peptide*

Suppl. Data 3: *Method parameters of PRM analysis*

Suppl. Data 4: *Light to heavy ratios of targeted peptides used for the assessment of regulated ubiquitination sites in EGTA -vs- Ca2+ treated synaptosomes by PRM.*

Suppl. Data 5: *Light to heavy ratios of targeted CaMKIIa peptides used for the assessment of changes in K291 ubiquitination and T286 phosphorylation of CaMKIIα in EGTA -vs- Ca2+ treated synaptosomes by PRM.*

Suppl. Data 6: *Light to heavy ratios of targeted peptides used for the assessment of changes in K291 ubiquitination and T286 phosphorylation of CaMKIIα in DMSO-vs-ionomycin treated HeLa (Kyoto) cells.*

4. References

1. Cox, J., Neuhauser, N., Michalski, A., Scheltema, R. A., Olsen, J. V., and Mann, M. (2011) Andromeda: A Peptide Search Engine Integrated into the MaxQuant Environment. *J. Proteome Res.* 10, 1794–1805

2. Tyanova, S., Temu, T., and Cox, J. (2016) The MaxQuant computational platform for mass spectrometry-based shotgun proteomics. *Nat. Protoc.* 11, 2301–2319

3. The UniProt Consortium (2019) UniProt: a worldwide hub of protein knowledge. *Nucleic Acids Res.* 47, D506–D515

4. Chao, L. H., Stratton, M. M., Lee, I.-H., Rosenberg, O. S., Levitz, J., Mandell, D. J., Kortemme, T., Groves, J. T., Schulman, H., and Kuriyan, J. (2011) A Mechanism for Tunable Autoinhibition in the Structure of a Human Ca2+/Calmodulin- Dependent Kinase II Holoenzyme. *Cell* 146, 732–745

5. Notredame, C., Higgins, D. G., and Heringa, J. (2000) T-coffee: a novel method for fast and accurate multiple sequence alignment 1 1Edited by J. Thornton. *J. Mol. Biol.* 302, 205–217

6. Silbern, I., Pan, K.-T., Fiosins, M., Bonn, S., Rizzoli, S. O., Fornasiero, E. F., Urlaub, H., and Jahn, R. (2021) Protein Phosphorylation in Depolarized Synaptosomes: Dissecting Primary Effects of Calcium from Synaptic Vesicle Cycling. *Mol. Cell. Proteomics* 20, 100061

7. Rosenberg, O. S., Deindl, S., Sung, R.-J., Nairn, A. C., and Kuriyan, J. (2005) Structure of the Autoinhibited Kinase Domain of CaMKII and SAXS Analysis of the Holoenzyme. *Cell* 123, 849–860

8. Rellos, P., Pike, A. C. W., Niesen, F. H., Salah, E., Lee, W. H., Delft, F., and Knapp, S. (2010) Structure of the CaMKIIδ/Calmodulin Complex Reveals the Molecular Mechanism of CaMKII Kinase Activation. *PLoS Biol.* 8, e1000426

9. Myers, J. B., Zaegel, V., Coultrap, S. J., Miller, A. P., Bayer, K. U., and Reichow, S. L. (2017) The CaMKII holoenzyme structure in activation-competent conformations. *Nat. Commun.* 8, 15742
